# CMUT-Based Sensor for Acoustic Emission Application: Experimental and Theoretical Contributions to Sensitivity Optimization

**DOI:** 10.3390/s21062042

**Published:** 2021-03-14

**Authors:** Redha Boubenia, Patrice Le Moal, Gilles Bourbon, Emmanuel Ramasso, Eric Joseph

**Affiliations:** Department of Applied Mechanics, CNRS/UFC/ENSMM/UTBM, FEMTO-ST Institute, University Bourgogne Franche-Comté, 25000 Besancon, France; patrice.lemoal@femto-st.fr (P.L.M.); gilles.bourbon@femto-st.fr (G.B.); emmanuel.ramasso@femto-st.fr (E.R.); ejoseph@univ-fcomte.fr (E.J.)

**Keywords:** sensor, capacitive micromachined ultrasonic transducer, sensitivity, modeling, characterization, acoustic impedance

## Abstract

The paper deals with a capacitive micromachined ultrasonic transducer (CMUT)-based sensor dedicated to the detection of acoustic emissions from damaged structures. This work aims to explore different ways to improve the signal-to-noise ratio and the sensitivity of such sensors focusing on the design and packaging of the sensor, electrical connections, signal processing, coupling conditions, design of the elementary cells and operating conditions. In the first part, the CMUT-R100 sensor prototype is presented and electromechanically characterized. It is mainly composed of a CMUT-chip manufactured using the MUMPS process, including 40 circular 100 µm radius cells and covering a frequency band from 310 kHz to 420 kHz, and work on the packaging, electrical connections and signal processing allowed the signal-to-noise ratio to be increased from 17 dB to 37 dB. In the second part, the sensitivity of the sensor is studied by considering two contributions: the acoustic-mechanical one is dependent on the coupling conditions of the layered sensor structure and the mechanical-electrical one is dependent on the conversion of the mechanical vibration to electrical charges. The acoustic-mechanical sensitivity is experimentally and numerically addressed highlighting the care to be taken in implementation of the silicon chip in the brass housing. Insertion losses of about 50% are experimentally observed on an acoustic test between unpackaged and packaged silicon chip configurations. The mechanical-electrical sensitivity is analytically described leading to a closed-form amplitude of the detected signal under dynamic excitation. Thus, the influence of geometrical parameters, material properties and operating conditions on sensitivity enhancement is clearly established: such as smaller electrostatic air gap, and larger thickness, Young’s modulus and DC bias voltage.

## 1. Introduction

Acoustic emission (AE) is a nondestructive technique used in a structural health monitoring (SHM) technique and material characterization. The technique relies on receivers to detect elastic waves generated by a change in the structural integrity [1]. The elastic waves involved are characterized by amplitudes in the nanometer range [2], therefore, monitoring structures by AE requires sensors with a suitable sensitivity and signal-to-noise ratio [3].

Up to now, the detection of acoustic emissions has usually been performed by piezoelectric sensors [4] because of their important feedback from applications in the field of nondestructive testing, either as transmitter or receiver. Their characteristics intrinsically give them a limited frequency bandwidth and an impedance mismatch with respect to the wave propagation medium of the waves (typically, 35 MRayls for piezoelectric against 17 MRayls for aluminum and about 2 MRayls for the coupling material). However, these problems are partly solved by the addition of a backing material improving the sensitivity and enlarging the bandwidth [5,6] and front-side matching layers to adapt the acoustic impedance of the tested materials.

Capacitive micromachined ultrasonic transducers (CMUTs) can be an alternative to piezoelectric sensors [7,8], particularly because they benefit from the advantages of microelectronics: ease of mass production, miniaturization, flexibility and therefore, integration into complex devices and different topologies. Acting like microphones, CMUTs are capacitive membranes that vibrate under dynamic excitations (as acoustic waves). The mechanical vibrations induce capacitance variations and thus measurable electrical currents. CMUTs are generally characterized by a large bandwidth and low impedance making them well-adapted to acoustic emission applications. However, the low signal-to-noise ratio and sensitivity are clearly the weak points of CMUTs that are tackled in several publications.

About CMUT design, theoretical studies have shown the influence of electrode size on their efficiency. Indeed, the size of the electrodes acts on the parasitic capacitance, hence on the sensitivity and bandwidth of CMUT [9]. Membrane configuration can also be an important study parameter to increase the performance of CMUT. Manufactured by a wafer bonding process, Huang et al. [10] compared three different membrane configurations (rectangular, tent and square) at a bias voltage of 20 V. The two configurations tent and rectangular were found to have higher sensitivity than the square one (65% and 44%, respectively) in received mode.

Concerning the configuration of electrical connections, Cheng et al. [11] presented a solution of electrical interconnections reducing the parasitic capacitance from 2.75 pF to 1.5 pF, thus improving the efficiency of CMUT. To reduce noise, Gurun et al. [12] removed the wire bonding and the bonding pad between the CMUT and the amplifier. Indeed, this is because the wiring and the bonding pad add parasitic capacitance, which increases noise and decreases the sensitivity of the CMUT.

Regarding the conversion of mechanical vibrations into an electrical signal, Wright [13] optimized the sensitivity of a MEMS sensor by designing a new transimpedance amplifier (LMV 972). This amplifier increased the signal-to-noise ratio from 8.4 [*V*/*V*] to 41.2 [*V*/*V*] for the MEMS sensor, which remained low compared to the signal-to-noise ratio of piezoelectric sensors (475 [*V*/*V*]).

Especially for AE applications, Ozevin et al. [14] developed a narrow-band CMUT manufactured by MUMPs. The proper functioning of the CMUT required a vacuum package for better sensitivity. Ozevin et al. [15] improved their previous CMUT by increasing the active area of the CMUT (from 2.51 mm^2^ to 6.97 mm^2^) and thus its sensitivity allowing the sensor to operate at atmospheric pressure. Using six independent transducers, Ozevin et al. [16] developed capacitive MEMS covering a frequency range 100 kHz to 500 kHz. For the 100-unit cells, the maximum detected amplitude to the response of the pencil lead breaking on the ceramic package is 0.05 volt, which remains a low-level sensitivity.

Saboonchi and Ozevin [17] have compared MEMS AE transducers manufactured by an electroplating technique with piezoelectric transducers having a similar frequency range (50–200 kHz). The results of their experiment showed the good sensitivity of capacitive MEMS AE transducers with a signal-to-noise ratio close to piezoelectric sensors (34.42 dB for MEMS-S1 vs. 42.65 dB for piezoelectric R6) and better than piezoelectric at the central frequency (58.76 dB for MEMS-S1 vs. 54.66 dB for piezoelectric R6). However, the transducers are sensitive to a single wave direction, which can be disadvantageous in case of damage inside the materials.

In a previous article [7], we presented the application potential of a CMUT-based AE sensor realized by the design of a first version of the sensor (called CMUT-V1 in the following) manufactured using the polyMUMPS surface micromachining process. This previous work focused more particularly on two positive aspects: the multifrequency aspect involving different individual membranes and the bandwidth aspect including the intrinsic capabilities of an array of 9 identical membranes.

This paper proposes to study how to improve the signal-to-noise ratio and especially the sensitivity of these sensors by various potential means such as design, packaging, signal processing and structure-sensor coupling. In Section 2, a second version of the sensor, fabricated with the same manufacturing MUMPS process and named CMUT-R100 in the following, is presented including practical modifications in design, electrical connections and packaging. Experimental tests show a significant increase in sensitivity and in turn, in the signal-to-noise ratio. Therefore, a classical frequency filtering method is applied to show the interest of designing hardware solutions that achieve this filtering. In Section 3, the overall sensor sensitivity is divided into an acoustic-mechanical part and a mechanical-electrical one. The acoustic-mechanical contribution mainly determined by the monitored structure-sensor coupling interface is studied according to theoretical and experimental reflection elements. The mechanical-electrical contribution defined by the relation between the mechanical vibration of the CMUT membrane and the resulting electrical charges is theoretically evaluated in a general way and according to the amplifier used. Lastly, key design parameters in terms of dimensions and the constituent materials are outlined and trends for future works are suggested.

## 2. Design, Packaging and Experimental Characterization of the CMUT-R100 Sensor

### 2.1. Design and Packaging

The principle of AE detection by CMUT-based sensors is briefly recalled. When a structure is damaged, stress waves are released and propagate in it. These elastic waves are transferred to the sensor through the structure-sensor interface, causing the CMUT membranes to vibrate and thus generate an electric current by capacitance changes.

**Figure 1:**
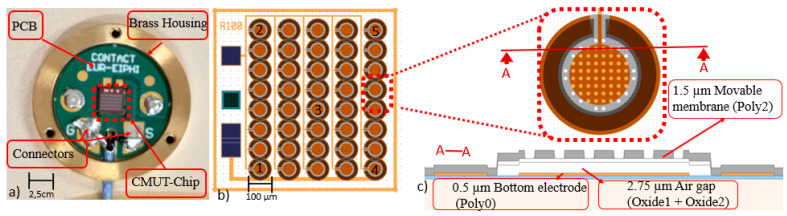
(**a**) CMUT-R100, (**b**) CMUT-chip layout top view and (**c**) sectional views of the CMUT elementary cell.

Figure 1a shows the CMUT-R100 (without top cap) consisting of a CMUT-chip with 40 elementary cells (see Figure 1b,c) wire bonded to a PCB, all placed in a brass housing.

The elementary cell of the CMUT-chip was a movable polysilicon membrane (see Figure 1c) with the following dimensions and material properties: radius *R* of 100 µm, thickness *t* of 1.3 µm (this was the measured value which was different from the “manufacturer” value of 1.5 µm, as shown in Figure 1c), Young’s modulus *E* of 160 GPa, Poisson’s ratio *ν* of 0.22 and density *ρ* of 2330 kg.m^−3^. Under dynamic excitations, this membrane was able to vibrate above an air cavity of 2.1 µm height (this was also the measured value to be compared to 2.75 µm shown in Figure 1c) defining a capacitance between bottom (Poly 0 layer) and top (Poly 2 layer constituting the membrane) electrodes. The capacitance variations that caused the measured electrical current required a DC voltage applied between the bottom and top electrodes. As shown on Figure 1c, holes had to be etched for the membranes releasing and their configurations (68 air-filled cavities with 10 µm diameter and 30 µm pitch [7,18]) in the case of 100 µm radius membranes allowed the coverage of a quite large frequency range between 310 kHz and 420 kHz. Further information on the steps of the manufacturing process is given in [7].

Table 1 reports the size differences between CMUT-R100 and CMUT-V1. Contrary to the previous version, the CMUT-chip was dedicated to only one type of radius in order to minimize parasitic crosstalk and increase similar contributions. On the other hand, the chip and packaging areas were respectively divided by 4 and 3, which reduced the sensor footprint on the monitored structure. The CMUT-chip element was connected on printed circuit board (PCB) for electrical connection, via a gold wire bonding and was housed in a brass cylinder 16 mm in diameter and 1.6 mm high (see Figure 1a).

**Figure 2:**
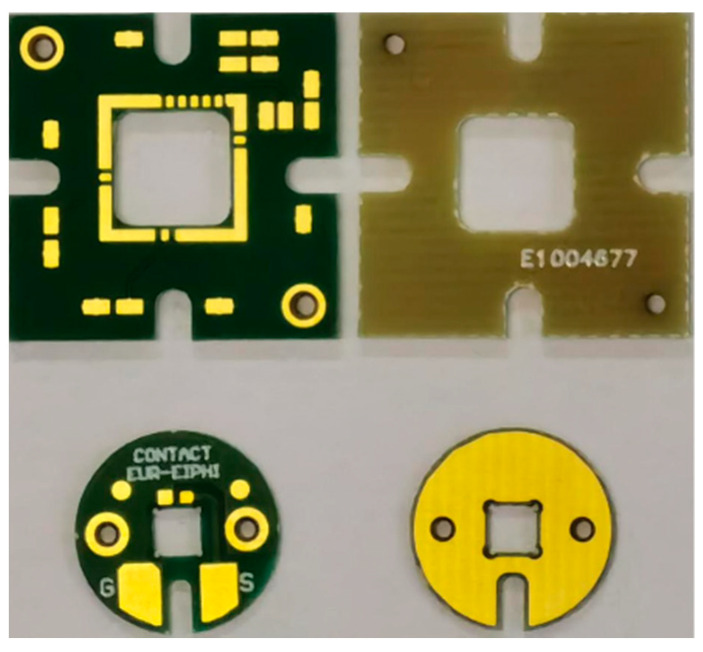
Top and bottom view of PCB versions of CMUT-V1 (square/at the top) and CMUT-R100 (circular/at the bottom).

Special attention was paid to the PCB design to increase reliability by reducing parasitic signals, as well as electromagnetic disturbances. The first step was to reduce the risk of interference from one component to another by increasing the width of the outer layer tracks and the isolation distance (from 0.15 mm to 0.25 mm) and by reducing the number of signal contacts (from 6 to 1, see Figure 2). Indeed, the new version was composed of only 100 µm radius and therefore required only one bias voltage, whereas the first version was composed of six different radii (50 µm, 75 µm, 100 µm, 150 µm, 200 µm and 250 µm) and required as many bias voltages. The second step was to reduce the capacitance effect by reducing the thickness of the PCB (from 1 mm to 0.5 mm) to increase the distance between the PCB and the top and by increasing the number of layers (from 1 to 2) to avoid coupling between the top of PCB and the PCB support. Figure 2 shows the top and bottom view for the first version (square design) and the new version (circular design) of the PCB.

In addition, the connection wires were slightly modified. An inner conductor of 0.2 mm (vs. 0.3 mm) with a nominal capacitance of 85 pF/m (vs. 100 pF/m) and an attenuation below than 115 dB/100 m (vs. 118 dB/100 m) at 400 MHz was chosen to reduce electrical disturbances and possibly increase the CMUT sensitivity.

### 2.2. Experimental Characterization

#### 2.2.1. Bias Voltage, Resonance Frequency and Bandwidth of the CMUT Cell

This section reviews the optimum operating range of the CMUT according to its frequency band and V_DC_ bias voltage. The pull-in voltage controlled the maximum voltage before short-circuiting (in the absence of insulating layers which is the case) and was therefore critical for the CMUT. For the electrical characterization, five elementary cells were tested on the CMUT-chip. They were considered at different locations (see Figure 1b) to validate the homogeneity of the manufactured chip. A V_DC_ bias voltage was applied to the CMUT-chip with the Keysight B2987A electrometer via two microprobes between 0 V and the pull-in voltage.

**Figure 3:**
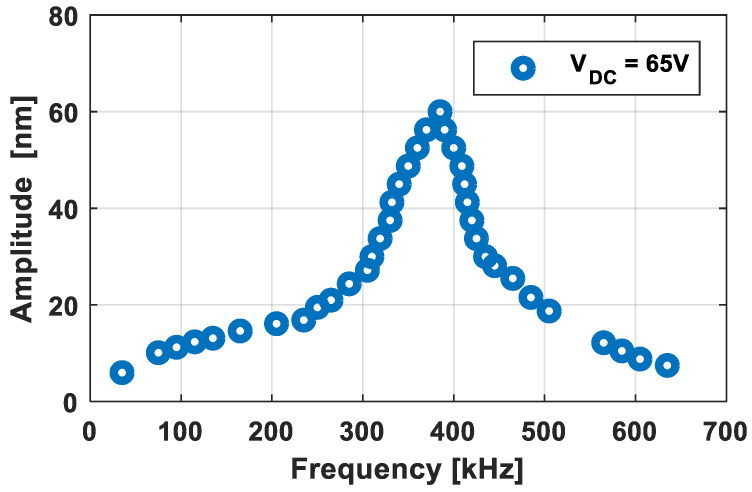
Maximum vibration amplitude as a function of the frequency at 65 V bias voltage.

Using a synthesizer function generator (Helwett Packard 3325 B), a 0.5 V peak-to-peak V_AC_ alternating voltage was superimposed on the DC bias voltage sweeping the frequency range between 50 kHz to 700 kHz. Figure 3 reports the maximum vibration amplitude of an elementary cell as a function of the scanning frequency using Polytec laser Doppler vibrometer. The experimental collapse voltage was estimated to be around 85 V_DC_.

**Figure 4:**
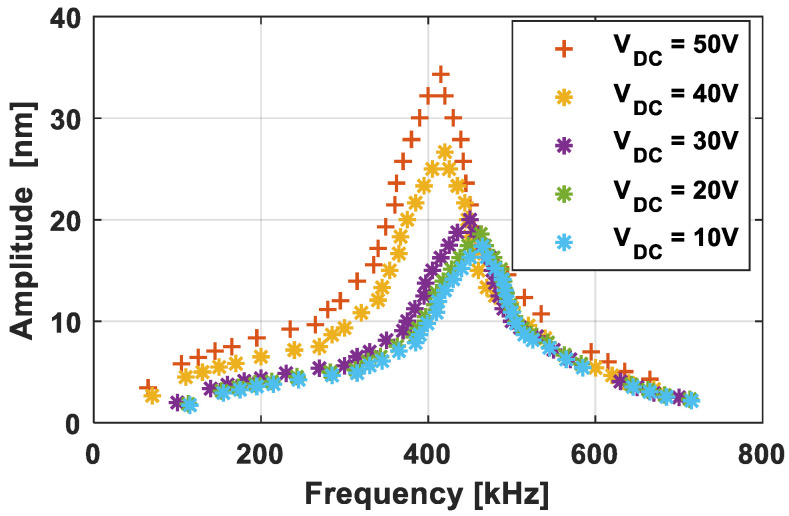
Maximum vibration amplitude as a function of the frequency for DC bias voltage in the range 10–50 V_DC_.

It will be discussed in Section 3 that the sensitivity is proportional to the ratio between the DC bias and the pull-in voltages. On the other hand, short-circuit risks become high when the DC bias and the pull-in voltages are very close. Therefore, a DC bias voltage was chosen at about 80% of the pull-in value, i.e., 65 V, to study the first frequency response proposed in Figure 4. This was obtained by averaging the responses of five elementary cells with measurement errors of 5.3%. At 65 V_DC_ (see Figure 3), the resonant frequency and the −3 dB bandwidth were respectively accessed at f_r_ = 385 kHz and Δf = 110 kHz (from f_1_ = 310 kHz to f_2_ = 420 kHz) leading to a quality factor Q = f_r_/Δf of 3.5. Figure 4 shows the frequency response for five additional voltages (from 10 V_DC_ to 50 V_DC_ with a 10 Volt step). As expected due to the electrostatic softening, the resonant frequency decreased with the DC bias voltage (i.e., 465 kHz at 10 V_DC_ and 385 kHz at 65 V_DC_). However, the bandwidth of −3 dB was fairly constant around 110 kHz. Thus, the quality factor was relatively independent of the bias voltage around 3.5.

#### 2.2.2. Electro-Acoustic Characterization: CMUT-R100 vs. CMUT-V1

**Figure 5:**
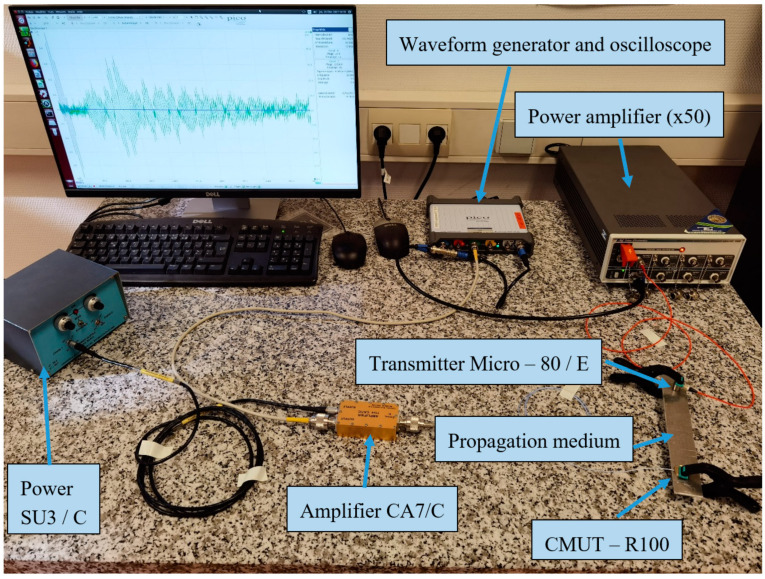
Setup of the electro-acoustic characterization.

The electro-acoustic characterization consisted of measuring the electrical response of the CMUT sensor to a broadband acoustic emission. The materials and methods used in [7] were again applied in the present work to compare the acoustic performances of CMUT–R100 and CMUT–V1 in terms of elastic wave detection. A Micro-80/E ultrasonic piezoelectric transducer from the Mistras Group Ltd. with a diameter of 9 mm and a height of 11 mm was used as a transmitter to generate acoustic waves propagating in an aluminum beam (30 mm wide, 200 mm long and 3 mm high). This piezoelectric transducer had an operating frequency range of 200–900 kHz and was driven by a 300 kHz center frequency signal. A 6-cycle windowed sine wave excitation signal centered at 300 kHz was applied using a Picoscope 4825 waveform generator. This electric signal was amplified by Tabor Electronics 9100A with a fixed gain of 50. The Cooknell SU3/C and CA7/C gain charge amplifier between the Picoscope and the CMUT sensors had two functions: the application of the DC bias voltage and the amplification of the induced electric charges at the terminals of the CMUT cells. Figure 5 shows the experimental set-up for the electro-acoustic characterization of CMUT-R100 and CMUT-V1 sensors using the Micro-80/E transmitter. In the present study, we compared new and old versions of the CMUT sensors (CMUT-R100 and CMUT-V1) to highlight the improvements in sensitivity achieved whereas in [7] the authors compared the CMUT-V1 to Micro80/R to illustrate the abilities of CMUT sensors.

**Figure 6:**
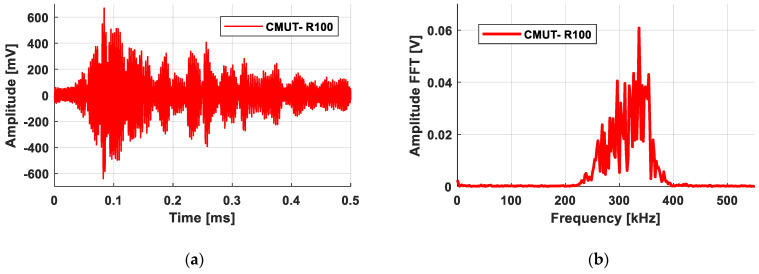

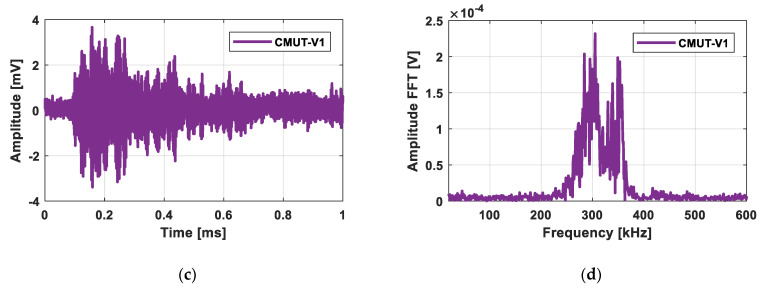
Time signals recorded by (**a**) CMUT-R100, (**c**) CMUT-V1, and corresponding FFT of time signals (**b**) for CMUT-R100 and (**d**) for CMUT-V1.

Figure 6 shows that the received time signals had similar shapes with a much higher sensitivity for CMUT-R100. The amplitude of the transient signal was almost 200 times larger, i.e., 700 mV to 3.7 mV. Fast Fourier transforms (FFT) of time signals were slightly different with a vibrational energy which seemed to be more around 340 kHz for CMUT-R100. This signal amplification could be partly attributed to the increase in the number of cells from 9 to 40, to the improvement of the electrical packaging (connections and PCB), and to the reduction of parasitic crosstalk (CMUT-chip dedicated to one cell type). On the other hand, the amount of noise had increased from 0.5 mV to 50 mV in the same time. The increase in sensitivity could in turn lead to an increase in the amount of noise. Thus, the signal-to-noise ratio remained improved going from 17 dB to 23 dB. However, this gain in sensitivity made it possible to envisage postprocessing of the signal in order to go further.

#### 2.2.3. Signal Processing

The objective of this section was to improve the signal-to-noise ratio of the last measurement. To achieve this objective, a low pass filter was coded on Matlab. A Butterworth filter was chosen for its ease of implementation. Indeed, knowing the transfer function of the filter, the filter can be electronically realized by the Cauer method. The Butterworth filter is linear with a transfer function module (Gain) at order *n* defined by [19]:

(1) $$\left|H_{n}\left(j\omega \right)\right|=\frac{1}{\sqrt{1+{\left(\omega /\omega_{c}\right)}^{2n}}},$$

with ω=2πf and $\omega_{c}=2\pi f_{c}$, $f_{c}$ represents the cutoff frequency at −3 dB.

**Figure 7:**
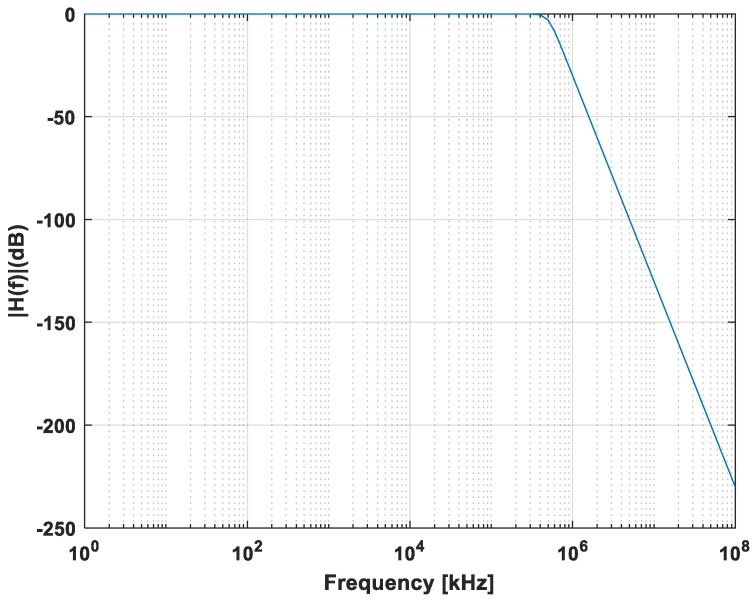
Low-pass filter gain.

In this context, a 5th order Butterworth filter with a cutoff frequency of 500 kHz at −3 dB is considered. This gives a linear response with a decrease of 100 dB per decade. The gain of the transfer function of our filter is shown in the following Figure 7.

**Figure 8:**
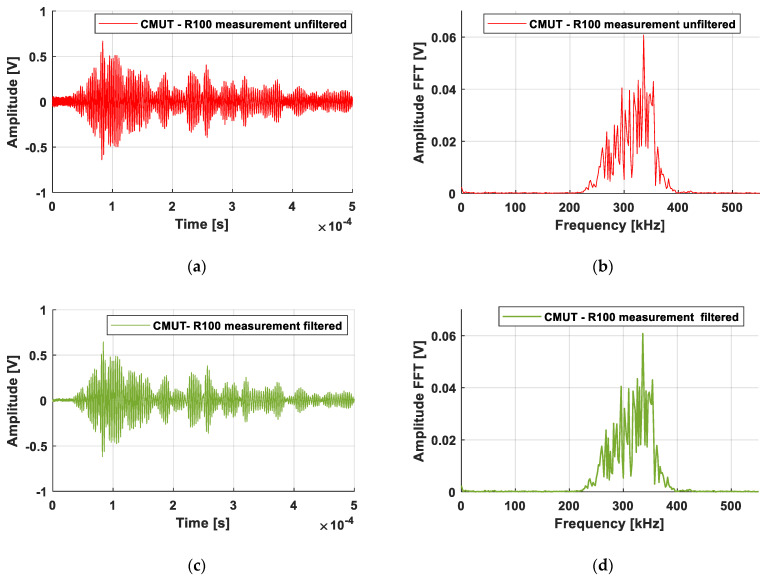
CMUT-R100 signals (**a**) unfiltered, (**c)** filtered and FFT (**b**) unfiltered, (**d**) filtered.

The time signals and their fast Fourier transform (FFT) were studied before and after the application of the filter on the detected CMUT-R100 signal (see Figure 8). Figure 8b,d confirmed a frequency response unaffected by the filter. Moreover, Figure 8a,c show that the unfiltered and filtered signals had the same amplitude with reduced noise for the postprocessed signal. Indeed, the noise amplitude of the received signal after filtering was five times lower than that of the unfiltered signal (10 mV vs. 50 mV before filtering). Finally, the signal-to-noise ratio reached 37 dB against 23 dB without processing. This proved the interest of designing hardware solutions that achieve this numerical filtering.

## 3. Sensor Sensitivity: Acoustic–Mechanical and Mechanical–Electrical Contributions

The overall sensitivity of the CMUT sensor can be broken down into two contributions as follows:

(2) $$\frac{\Delta Q}{\Delta W_{inc}}=\frac{\Delta Q}{\Delta W_{0}}\times \frac{\Delta W_{0}}{\Delta W_{inc}}=S_{mech\_elec}\times S_{acoust\_mech},$$

With respectively ΔQ, $\Delta W_{inc}$ and $\Delta W_{0}$ the variations of electrical charges, the variations of amplitude of the incident wave and the variations of amplitude of the membrane vibrations.

### 3.1. Acoustic-Mechanical Sensitivity

The acoustic-mechanical contribution reflects the ability of an elementary cell to vibrate in response to an incident wave. This is directly related to all potential losses, including the nature of the coupling at the different interfaces, i.e., the substrate/CMUT sensor and CMUT sensor/elementary cell interfaces and the quality factor of the membrane. The first two points concerning the energy transfer of the wave at each interface are examined in the following section through simulations and various experimental data. The quality factor mainly depends on the surrounding environment, which is assumed to be air in this context. For this reason, it is not analyzed in this paper but further works may be interested in the influence of geometrical shapes, dimensions and boundary conditions on the quality factor.

#### 3.1.1. Modeling

The decrease in the amplitude of the incident wave is related to two phenomena:

- attenuation characterized by α [dB/cm]
- insertion losses characterized by the reflection or transmission coefficients in terms of amplitude *r*_12_ or *t*_12_ or in terms of energy *R* or *T* at the interface between two media 1 and 2. An impedance measurement system for piezoelectric array element transducers:
  (3) $$r_{12}=\frac{Z_{1}-Z_{2}}{Z_{1}+Z_{2}},\;t_{12}=\frac{2Z_{1}}{Z_{1}+Z_{2}},\;R=r_{12}^{2}\;and\;T=1-R,$$
  with Z=ρ×v the acoustic impedance of the medium, ρ the density of the propagation medium, and v the wave velocity in the medium.

Acoustic coupling is ensured when the incident wave corresponds to that generated during an acoustic emission. In this condition, the transmission coefficient R is equal to zero and the attenuation is assumed to be zero. These two conditions are not available in a real situation. It was, therefore, important to carry out a theoretical study to show in our case the influence of insertion losses on the incident wave for each interface.

Different approaches allow the propagation of an elastic wave to be described over a succession of various material layers. Based on finite element calculations [20], the KLM equivalent circuit [21] or the Brekhovskikh iterative calculation [22], these methods aim to evaluate the acoustic impedance resulting from the crossing of the layers and the interfaces between these layers. In this work, the Brekhovskikh iterative method was used to determine the global coefficient of transmission and reflection. These coefficients result from the calculation of the equivalent acoustic impedance of the layered structures based on the successive application of the following Equation (4):

(4) $$Z_{n}=Z_{n}\left(\frac{Z_{n+1}-iZ_{n}\tan k_{n}x_{n}}{Z_{n}-iZ_{n+1}\tan k_{n}x_{n}}\right),$$

where ***Z_n_*** is the input impedance of any layer *n*, *Z_n_* is the acoustic impedance of the layer *n* material, *k_n_* is the wave number of the layer *n* (the ratio between the angular frequency pulsation and the wave velocity) and *x_n_* is thickness of layer *n*. The layered structures correspond to the possible coupling conditions encountered on the CMUT-R100 sensor.

#### 3.1.2. Influence of the Coupling Conditions on the Amplitude of the Detected Signal

In accordance with acoustic emission application, three configurations of coupling conditions were considered for this purpose:

- Case 1: the CMUT-chip is glued directly with araldite onto the brass housing which is coupled to the aluminum sample by a coupling gel supposed to be perfect (Si-Araldite-Brass/Al)
- Case 2: the CMUT-chip is glued directly with araldite to the aluminum sample (Si-Araldite-Al).
- Case 3: the configuration is similar to Case 1 with a defect in the air layer between the araldite and brass housing (Si-Araldite-Air-Brass/Al).

**Figure 9:**
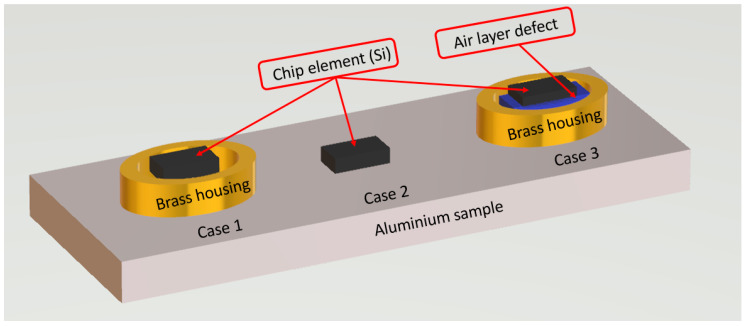
Three configurations of coupling conditions.

Case 3 (see Figure 9) is quite realistic because it is difficult to exert a sufficient bonding pressure between the CMUT-chip and the brass housing due to the very small size of the chip and the packaging.

**Figure 10:**
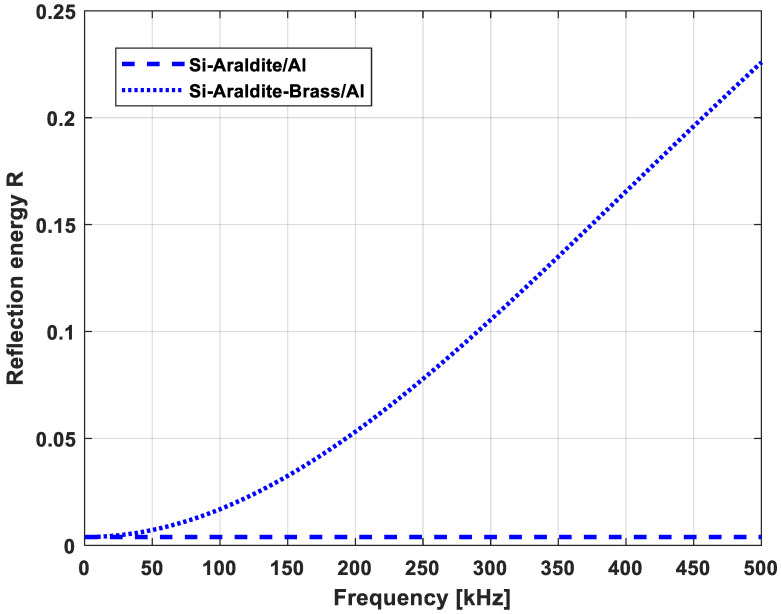
Reflection coefficient as a function of frequency for Case 1 (**…**) and Case 2 (**---**).

The working frequency band included the bandwidth of the CMUT-R100 (310–420 kHz) and the properties of each material used in the simulations are presented in Table 2 [23]. The evolution of the reflection coefficients as a function of frequency for Cases 1 and 2 is proposed in Figure 10. Case 1 showed a very low reflection coefficient consistent with the micrometer thickness of the araldite and the acoustic impedances of silicon and aluminum of the same order of magnitude. However, the brass layer characterized by a millimeter thickness and an impedance mismatch with other layers implied significant losses increasing with the working frequency.

**Figure 11:**
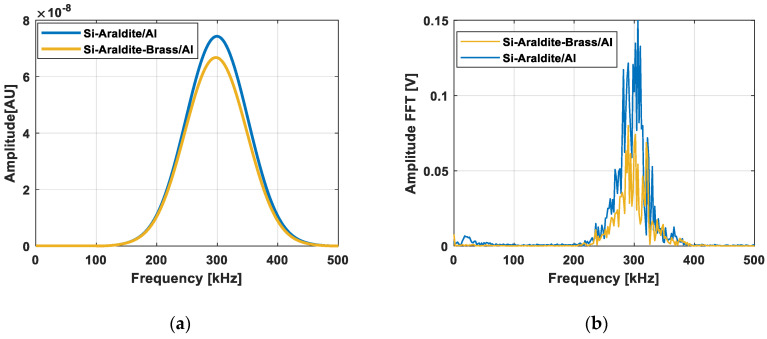
Amplitude of the transmitted signal as a function of frequency: (**a**) simulation and (**b**) experimental results.

**Figure 12:**
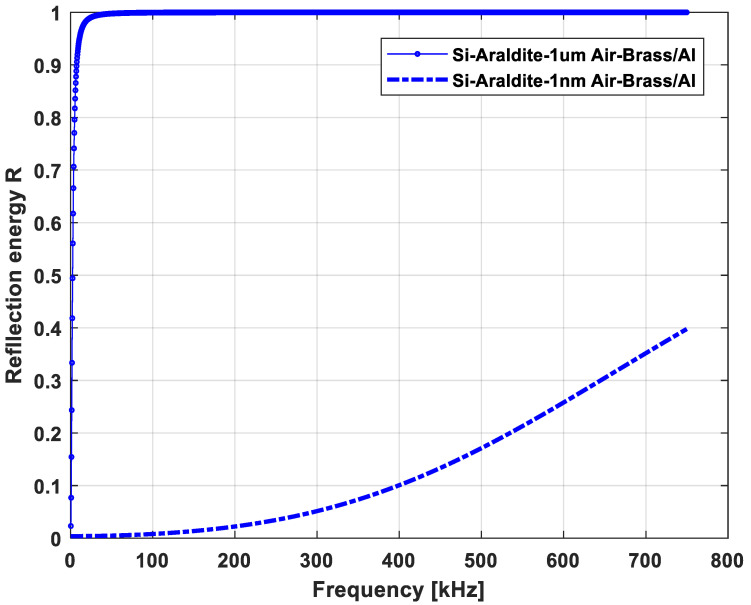
Influence of the air defect layer (1 nm and 1 um) on the reflection coefficient.

The amplitude of the transmitted as a function of frequency is shown in Figure 11. In Figure 11a, a virtual windowed sinusoid centered at 300 kHz with an arbitrary bandwidth was affected by the calculated transmission coefficient and in Figure 11b, the experimental data are reported. The attenuation between Case 1 and Case 2 of the detected signal was much greater for experimental data than for the theoretical evaluations, i.e., about 53% against 13%. The difference between the simulation and experimental results could be explained by the introduction of other defects such as material attenuation, air defect, and so on. In order to explain the influence of an air layer for example, Case 3 was considered with two air layer thicknesses of 1 nm and 1 µm. The simulation results presented in Figure 12 show that even the thinnest air layers could cause significant losses at the interfaces. Surely, an air layer of 1 µm characterized by an almost total reflection of the propagating wave was overestimated. However, air layers of 1 nm or 10 nm are quite realistic and cause energy losses.

Thus, control of the acoustic coupling between each material is important to reduce insertion losses and to maximize the amplitude of the transmitted signal. Special attention must be paid to the bonding of the components of the CMUT-R100 sensor and its coupling with the monitored structure by minimizing air layers as much as possible.

### 3.2. Mechanical-Electrical Sensitivity

The mechanical-electrical contribution is related to the capacity of the elementary cell to convert the mechanical vibration of the suspended membrane into electrical charges and thus into electrical current. This is the subject of the following analytical developments.

#### 3.2.1. Modeling

The capacitance C of the elementary cell can be expressed in terms of the initial electrostatic gap g and the total deflection w(r) including a DC part $w_{DC}\left(r\right)$ and an AC part $w_{AC}\left(r,t\right)$ according to [24]:

(5) $$C=\varepsilon_{0}2\pi \int_{0}^{R_{elec}}\frac{r}{\left(g-w\left(r\right)\right)}dr,$$

with $w\left(r\right)=w_{AC}\left(r,t\right)+w_{DC}\left(r\right)$ and where $\varepsilon_{0}$ and $R_{elec}$ are, respectively, the permittivity of vacuum or air, and the radius of the electrode (the radius of the electrode refers to the orange lower electrode in Figure 1b,c). The DC part $w_{DC}\left(r\right)$ is caused by the application of the DC bias voltage and the AC part $w_{AC}\left(r,t\right)$ is the vibration amplitude induced by the incident elastic waves.

Considering that $w_{AC}\left(r,t\right)\ll g-w_{DC}\left(r\right)$, the total capacitance $C_{tot}$ for *N_c_* cells is expanded to the first order in $\frac{w_{AC}\left(r\right)}{g-w_{DC}\left(r\right)}$, i.e.,:

(6) $$C_{tot}\approx N_{c}\varepsilon_{0}2\pi \int_{0}^{R_{elec}}\left(\frac{r}{\left(g-w_{DC}\left(r\right)\right)}+\frac{rw_{AC}\left(r,t\right)}{{\left(g-w_{DC}\left(r\right)\right)}^{2}}\right)dr,$$

The electrical current i generated by the mechanical vibration is:

(7) $$i=\frac{dQ}{dt}=\frac{d}{dt}\left(C_{tot}V\right)=V_{DC}\frac{dC_{tot}}{dt},$$

and thus,

(8) $$i\approx N_{c}V_{DC}\varepsilon_{0}2\pi \int_{0}^{R_{elec}}\frac{r}{{\left(g-w_{DC}\left(r\right)\right)}^{2}}\frac{dw_{AC}\left(r,t\right)}{dt}dr,$$

A harmonic form of the AC part is assumed: $w_{AC}\left(r,t\right)=W_{AC}\left(r\right)e^{i\omega t}$ with $W_{AC}\left(r\right)=W_{0}\frac{{\left(r^{2}-R_{m}^{2}\right)}^{2}}{R_{m}^{4}}$ as a fit in accordance with the actual boundary conditions; i.e., clamped at the membrane radius $R_{m}$ with a vibration amplitude at the membrane center $W_{0}.$

Thus, the electric current becomes:

(9) $$\left|i\right|\approx N_{c}\varepsilon_{0}2\pi \omega W_{0}V_{DC}\int_{0}^{R_{elec}}\frac{r}{{\left(g-w_{DC}\left(r\right)\right)}^{2}}\frac{{\left(r^{2}-R_{m}^{2}\right)}^{2}}{R_{m}^{4}}dr,$$

and finally, the mechanical-electrical sensitivity $S_{mech\_elec}$ can be written:

(10) $$S_{mech\_elec}\approx N_{c}\varepsilon_{0}2\pi V_{DC}\int_{0}^{R_{elec}}\frac{2\pi r}{{\left(g-w_{DC}\left(r\right)\right)}^{2}}\frac{{\left(r^{2}-R_{m}^{2}\right)}^{2}}{R_{m}^{4}}dr,$$

According to Equation (10), the mechanical-electrical sensitivity $S_{mech\_elec}$ is mainly controlled by the electrode and membrane radii, the electrostatic gap between the upper and lower electrodes, and the V_DC_ bias voltage.

The sensitivity of the Cooknell CA7 charge amplifier used, i.e., 250 mV/pC, defines the theoretical amplitude of the output voltage as follows:

(11) $$A\left(\mathrm{mV}\right)\approx 250.10^{12}N_{c}\varepsilon_{0}W_{0}V_{DC}\int_{0}^{R_{elec}}\frac{2\pi r}{{\left(g-w_{DC}\left(r\right)\right)}^{2}}\frac{{\left(r^{2}-R_{m}^{2}\right)}^{2}}{R_{m}^{4}}dr,$$

Thus, the amplitude of the detected signal A(mV) will preferentially be studied afterwards instead of the mechanical-electrical sensitivity considering one cell and a vibration amplitude $W_{0}$ of 1 nm, i.e., *A*(mV/nm/*N_c_*).

#### 3.2.2. Influence of the Surface Electrode on the Amplitude of the Detected Signal

**Figure 13:**
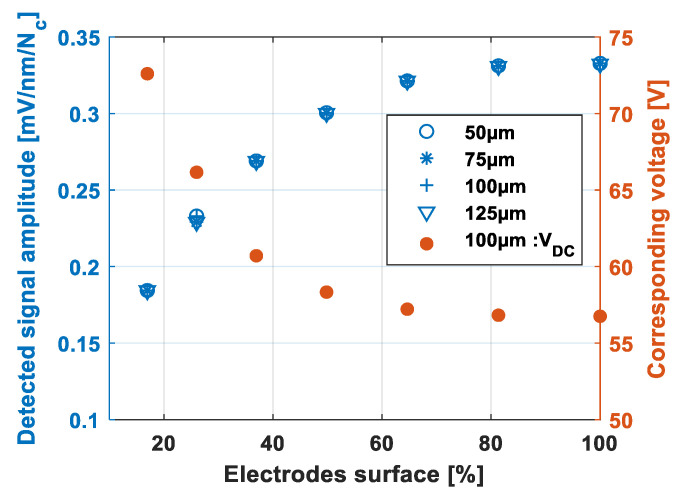
Influence of the electrode surface on the amplitude of the detected signal and the corresponding voltage.

In the studied configuration, the upper electrode consisted of the entire membrane made of a conductive layer “poly 2”. Only the bottom electrode deposited on the substrate and made of a conductive layer “poly 0” could be structured according to different radii. The electrode surface was given by the square of the ratio of the radius of the bottom electrode to that of the membrane. Based on the analytical developments of Nikoozadeh et al. [25], the pull-in voltage and the static deflection $w_{DC}\left(r\right)$ for a given $V_{DC}$ bias voltage were calculated with the dimensions and material properties described at Section 2.1. The chosen $V_{DC}$ bias voltage, referred to as the corresponding DC voltage in the following, was considered to be equal to 60% of the pull-in voltage, i.e., $V_{60\%}$. Numerical simulations were performed with different membrane radii 50 µm, 75 µm, 100 µm and 125 µm. Figure 13 shows that the evolution of the amplitude of the detected signal, which is to be read on the left-hand ordinate axis, did not depend on the membrane radius for a given electrode surface. This condition was not satisfied by the corresponding voltage. In Figure 13, referring to the right-hand ordinate axis, the corresponding DC voltage is shown only for the 100 µm radius membranes which were the components of the CMUT-R100 sensor. Thus, for the 100 µm radius membranes, the amplitude of the detected signal and the corresponding voltage increased and respectively decreased to asymptotic values around 0.33 mV and 57 V, respectively, from electrode surface values of the order of 65%, i.e., an electrode radius around 80% of that of the membrane. It can already be deduced that an electrode radius of 80% can be considered as the optimum value [26], because beyond this, only the parasitic capacitances increased. Considering the asymptotic value of the detected signal and that $w_{DC}\left(r\right)\ll g$, the amplitude of the detected signal can be evaluated for an electrodes surface of 100% by integration of Equation (11):

(12) $$A\left(\mathrm{mV}/\mathrm{nm}/N_{c}\right)\approx \frac{2\pi .250.10^{12}10^{-9}\varepsilon_{0}\alpha V_{PI}}{6}{\left(\frac{R_{m}}{g}\right)}^{2}\approx 2\;318.10^{-6}\times \alpha V_{PI}\times {\left(\frac{R_{m}}{g}\right)}^{2},$$

with *α* the fraction of the pull-in voltage $V_{PI}.$

#### 3.2.3. Influence of the DC Bias Voltage on the Amplitude of the Detected Signal

Considering the DC bias voltage as a fraction of the pull-in voltage, the first step is to determine the pull-in voltage according to the mechanical and geometrical parameters. Zhang et al. [27] developed an analytical model to calculate the pull-in voltage of a flat circular CMUT cell with a sealed cavity. In the present case, the CMUT cell is perforated, resulting in a pressure balance between the cavity and the surrounding medium. The formula (considering a pressure difference $p_{a}=0$) gives the voltage for the ratio $x=\frac{w_{max}}{g}$ as follows:

(13) $$V=\frac{8}{3}\sqrt{\frac{E}{\varepsilon_{0}\left(1-\nu^{2}\right)}}\frac{{\left(t\times g\right)}^{3/2}}{R_{m}^{2}}f\left(x\right),$$

with $f\left(x\right)=\sqrt{\frac{x^{2}}{\left(1/\left(1-x\right)-\tanh^{-1}\left(\sqrt{x}\right)/\sqrt{x}\right)}}.$

The pull-in voltage is obtained when *V* is maximal and hence when *f(x)* is maximal. As Max[f(x)]≈0.578 for x≈0.463

(14) $$V_{PI}\approx 1.540\sqrt{\frac{E}{\varepsilon_{0}\left(1-\nu^{2}\right)}}\frac{{\left(t\times g\right)}^{3/2}}{R_{m}^{2}},$$

Equation (14) has been validated by a very good correlation, i.e., a maximum relative error of 1.2%, with numerical simulations taking into account various mechanical properties and geometrical parameters (gap, thickness and membrane radius). Combining Equations (12) and (14), the amplitude of the detected signal for 1 nm of center vibration amplitude $W_{0}$ of the membrane can be written as:

(15) $$A\left(\mathrm{mV}/\mathrm{nm}/N_{c}\right)\approx 1.200\sqrt{\frac{E}{\left(1-\nu^{2}\right)}}\alpha \sqrt{\frac{t^{3}}{g}},$$

**Figure 14:**
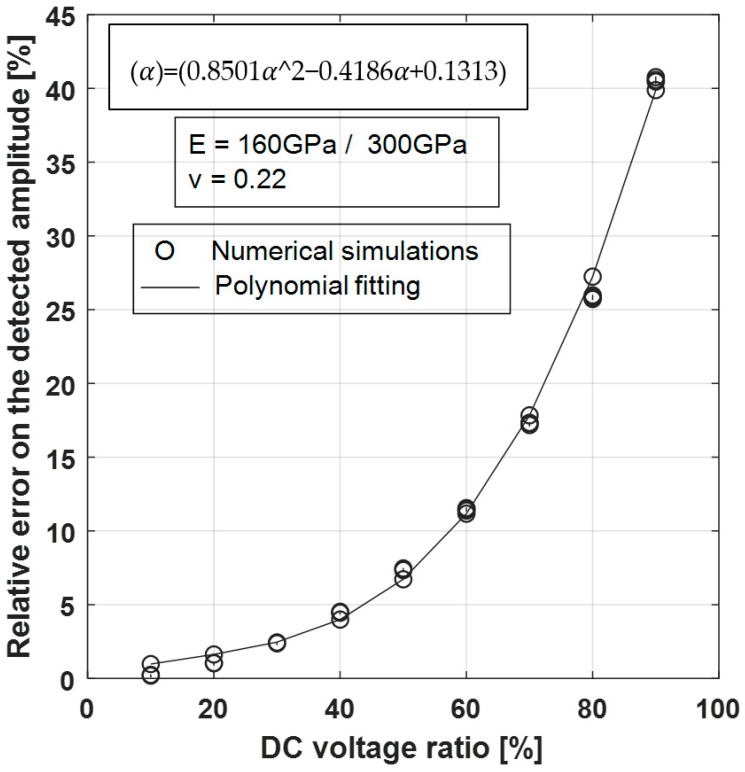
Relative error on the evaluated detected amplitude according to the DC voltage ratio (o) Numerical simulations and (-) polynomial fitting.

Equation (15) is based on the assumption that the static deflection $w_{DC}\left(r\right)$ is small relative to the electrostatic gap ***g***. However, this assumption is even less valid the closer the DC voltage is to the pull-in one; which is the case for the optimization of the sensor sensitivity. Figure 14 shows that the relative error on the amplitude of the detected signal exceeded 10% from a DC voltage ratio $\left(V_{DC}/V_{PI}\right)$ of 60%, reached 25% for 80% ratio and then 40% for 90% ratio.

A large set of points (i.e., 600 points per DC voltage ratio) was considered to scan the material properties (160 GPa for polysilicon and 300 GPa for silicon nitride) and geometrical parameters (gap from 0.5 µm to 5 µm, thickness from 0.5 µm to 3 µm and membrane radius from 30 µm to 120 µm) within a “realistic” range. It can be seen that the relative errors seemed to only depend on the DC voltage ratio; which made it possible to envisage a third-degree polynomial fitting. Thus, the amplitude of the detected signal can be accessed accurately with a relative error of less than 1.2% by the following analytical expression:

(16) $$A\left(\mathrm{mV}/\mathrm{nm}\right)\approx 1.200N_{c}\sqrt{\frac{E}{\left(1-\nu^{2}\right)}}\alpha \sqrt{\frac{t^{3}}{g}}\left(1+E\left(\alpha \right)\right),$$

with $E\left(\alpha \right)=\alpha \left(0.8501\alpha^{2}-0.4186\alpha +0.1313\right)$, *α* representing the DC voltage ratio.

A numerical application of Equation (16) considering 65 V_DC_ (80% of V_PI_), 40 elementary cells, geometrical parameters and material properties of the manufactured membranes gives for the acoustic emission test an amplitude of the detected signal about 20 mV for a vibration amplitude of 1 nm. Thus, the measured signal amplitude of about 700 mV leads by Equation (16) to about 35 nm vibration amplitude, which is a realistic value in this context.

#### 3.2.4. Optimization Ways: Trends for Future Works

According to Equation (16), the amplitude of the detected signal and thus the sensitivity of the sensor can be estimated in the dimensioning phase and some ways of optimization are highlighted as follows: smaller electrostatic gaps g, larger thicknesses *t*, a stiffener membrane material *E/(1−ν^2^)* and a higher DC voltage ratio α.

If the electrical parameter α was already studied for the CMUT-R100 sensor, the geometrical and material parameters cannot be modified so easily from a given micromanufacturing process. One the one hand, there is a small technological capacity for change: in the case of MUMPS process, the constituent material is polysilicon, with two possible polysilicon layers Poly 1 (2 µm) and Poly 2 (1.5 μm) and two possible oxide layers defining the gaps Oxide 1 (2 µm) and Oxide 2 (0.75 μm). On the other hand, each technological change in thickness or gap requires a new fabrication run. Only the in-plane dimensions, mainly the membrane radius, can be directly scanned by the modification of the mask design.

Thus, the optimization of sensitivity should be thought about comprehensively by investigating new manufacturing processes that can be the subject of future work in the longer term. These should aim at simultaneously optimizing material, geometrical and electrical parameters.

Concerning the material properties, silicon nitride is a good candidate to replace polysilicon. Silicon nitride is already involved in CMUT fabrication [28] and its material properties (similar Poisson’s ratio and higher Young modulus: around 0.25 and 300 GPa, respectively, [29]) could improve, according to Equation (16), the amplitude of the detected signal by a factor of ≈1.4 compared to polysilicon.

**Figure 15:**
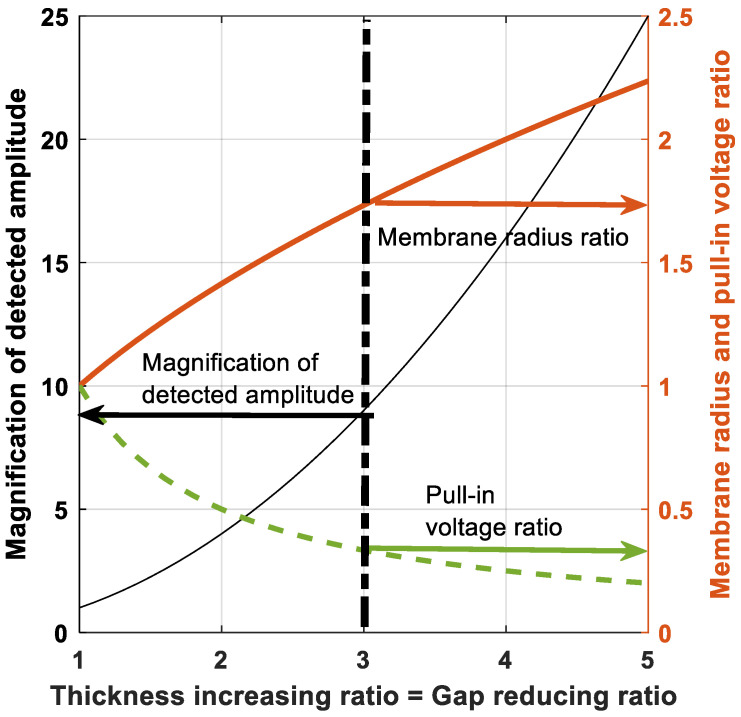
Influence of the thickness increasing/gap reducing ratio k on the detected amplitude, the membrane radius and the pull-in voltage.

To illustrate the possible magnification of the detected amplitude related to the geometrical parameters, it is assumed that the membrane thickness and the electrostatic gap can be affected by an inverse ratio; for example, the thickness is multiplied by *k* (increasing ratio) and the gap is divided by *k* (decreasing ratio). According to Equation (16), the magnification *M* evolves like the square of *k* (see Figure 15). Figure 15 highlights a realistic ratio of 3 (in this context, the thickness and the gap would be respectively about 4 µm and 0.7 µm) which leads to a detected amplitude 9 times higher. Furthermore, the resonant frequency *f* of a circular membrane is proportional to the thickness and inversely proportional to the square of membrane radius (Equation (1) in [7]). Thus, to maintain a given resonant frequency, the membrane radius must be modified according to the square root of the ratio *k*.

Lastly, according to Equation (14), the pull-in voltage is proportional to the product of the thickness and gap and inversely proportional to the square of the membrane radius. Thus, changing the membrane radius results in a reduction of the pull-in voltage by a factor equal to the ratio *k*. The evolutions discussed above and shown in Figure 15 can be summarized as follows:

(17) $$\{\begin{array}{c} \frac{e}{e^{ref}}=k\;\;\;and\;\;\;\frac{g}{g^{ref}}=\frac{1}{k}\Rightarrow M=\frac{A}{A^{ref}}=k^{2}\\ \frac{f}{f^{ref}}=1\;\;\;\;\;\;\;\;\;\;\;\;\;\;\;\;\;\;\;\;\;\Rightarrow \frac{R_{m}}{R_{m}^{ref}}=\sqrt{k}\\ \;\;\;\;\;\;\;\;\;\;\;\;\;\;\;\;\;\;\;\;\;\;\;\;\;\;\;\;\;\;\;\;\;\;\;\Rightarrow \frac{V_{PI}}{V_{PI}^{ref}}=\frac{1}{k} \end{array},$$

The parameters with “ref” in superscript correspond to the reference values of thickness, gap, detected amplitude, resonant frequency, membrane radius and pull-in voltage related to a given design and the resulting electromechanical characteristics.

## 4. Conclusions and Perspectives

In this paper, several lines of research have been undertaken to analyze and optimize the signal-to-noise ratio and especially the sensitivity of CMUT-based sensors dedicated to AE applications: the design, packaging and electrical connections of the sensor, the processing of the detected signal, the acoustic coupling conditions at the interfaces of the layered sensor structure and the design and operating conditions of the elementary cells.

The CMUT-R100 sensor based on previous works was developed, considering reduced sizes of the chip (2.5 mm × 2.5 mm) and the overall sensor (16 mm in diameter and 1.6 mm in height) and a higher number of elementary cells (40). The operating conditions in terms of DC bias voltage and frequency range of the elementary cells were respectively determined around 65 V and 310 kHz–420 kHz. The CMUT-chip was wire bonded on a PCB which was implemented in a brass housing to be tested on an aluminum sample instrumented by a piezoelectric transmitter simulating acoustic emission. The new design and packaging, the care taken to the electrical connections and the simple processing of the detected signal have contributed to increase the signal-to-noise ratio from 17 dB to 37 dB.

To go further, the sensitivity of the sensor was then analyzed in two parts: one acoustic-mechanical and other mechanical-electrical. Concerning the acoustic-mechanical part, experimental tests and the calculation of the acoustical impedance of layered structures showed the influence of insertion losses at the different interfaces.

It is essential to reduce to a minimum the layer thicknesses characterized by a poor matching of the acoustic impedance to the adjacent layers. In particular, air layers should be thinned as much as possible for example by exerting sufficient bonding pressure when bonding cannot be avoided.

The study of the mechanical-electrical part allowed the analytical definition of the amplitude of the detected signal from the geometrical parameters, the material properties, the operating conditions and the charge amplifier used. A first attempt to correlate the experimental amplitude of the detected signal and the closed-form solution gave a vibration amplitude of the CMUT cell of about 35 nm, where tens of nanometers were usually expected.

From Equation (16), the key elements of the design should be noted: the independence of the cell radius, design of a membrane with a large thickness and a small electrostatic gap, choice of a membrane material with a high Young modulus and operation at DC bias voltage as close as possible to the pull-in voltage. Membrane thickness, electrostatic gap and membrane material are parameters, which depend on the selected manufacturing process. For example, the MUMPS process has predefined steps with specific material layers and a too limited range of thicknesses and gaps. Thus, the study of a wider range of parameters leads to challenging work in the longer term as it requires the use of clean room microfabrication facilities to develop or codevelop inhouse manufacturing processes.

Future shorter-term work on this aspect will rather aim at extending the study of the mechanical-electrical sensitivity to other geometrical forms of membranes and possibly to other structures and/or boundary conditions. An aspect not treated in this context, the quality factor of vibrating membranes, is also a working perspective since it conditions the acoustic-mechanical sensitivity. The quality factor could depend on the geometrical shapes of membranes, the boundary conditions but also on geometrical parameters such as the radius and thickness of the membranes. Finally, regarding the practical aspects, the signal processing could be handled by a hardware solution for the CMUT-R100. On the other hand, other sensors as CMUT-R50, CMUT-R75 and CMUT-R150 could be designed and manufactured in the near future to cover a wider bandwidth from 150 kHz to 2000 kHz which is of practical interest for structural health monitoring.

## Figures and Tables

**Table 1:** CMUT-R100 vs. CMUT-V1.

	Number of Cells	Dimension of Chip [mm^2^]	Area and Volume of Packaging [mm^2^/mm^3^]
CMUT-V1	9	5 × 5	576/2304
CMUT-R100 version	40	2.5 × 2.5	201/322

**Table 2:** Material properties.

Material	Thickness [µm]	Density [Kg.m^−3^]	Wave Velocity [m.s^−1^]	Acoustic Impedance [MRayls]
Aluminum	3000	2700	6420	17.33
Brass	1000	8640	4700	40.6
Si	600	2330	8430	19.7
Air	1.0	1.2	344	0.429
Araldite	1.0	1160	2620	3.04
